# Metronomic chemotherapy for triple negative breast cancer?

**DOI:** 10.18632/aging.100947

**Published:** 2016-04-14

**Authors:** Teresa Di Desidero, Robert S. Kerbel, Guido Bocci

**Affiliations:** Dipartimento di Medicina Clinica e Sperimentale, Università di Pisa, Pisa, Italy

**Keywords:** metronomic chemotherapy, triple-negative breast cancer, topotecan, pazopanib, elderly patients

Breast cancer risk varies widely and increases with age. Despite the incidence for women older than 70 years being 33%, they are usually excluded from screening schedules and clinical trials. Furthermore, about 15% of breast cancers in older patients are of the triple-negative subtype (TNBC), which is known to be an aggressive histological subtype with very limited treatment options available, and associated with a very poor prognosis [1]. Indeed, the clinical management of elderly frail patients with TNBC can be complex and different from all the other breast cancer subtypes. Age-related factors such as comorbidities, deterioration of cognitive function, possible impairment in organ function and the concomitant use of other drugs, must be carefully assessed to avoid or minimize toxicity risks. In this context, low dose metronomic chemotherapy might represent a promising therapeutic option for elderly TNBC women [1]. Metronomic chemotherapy refers to the close, regular administration of conventional chemotherapy drugs at low, minimally toxic doses, with no prolonged break periods [2]. It has been shown to have an important stabilizing effect on cancer growth (including chemotherapy-resistant disease), to sometimes confer prolonged clinical benefits, and to have positive effects on the quality of life of patients with different types of cancer, by avoiding severe toxicities [3]. Moreover, the lower cost - when compared with the most standard chemotherapy regimens - and the oral administration (which reduces the need for hospitalization and enables patients to stay at home longer) are key characteristics of metronomic chemotherapy schedules, offering important advantages, especially for frail elderly patients with TNBC, for whom new therapeutic options are greatly needed. Quality of life benefits, oral administration of drugs and the possibility of spending more time at home are important characteristics of palliative treatments, as well as for maintenance or adjuvant therapies. Although, there are few clinical trials of metronomic therapy for elderly patients with breast cancer [1], the interest in assessing low-dose metronomic chemotherapy regimens in the clinic has been stimulated by the availability of a number of commercially available p.o. chemotherapy drugs, such as capecitabine, cyclophosphamide and vinorelbine. As an example, metronomic oral vinorelbine was investigated in at least two phase II trials resulting in good tolerability and safety profile in this elderly population with a satisfactory patient adherence to therapy [1].

Recently, a new preclinical schedule of metronomic topotecan combined with an antiangiogenic agent such as a tyrosine kinase inhibitor (i.e. pazopanib) was found to cause significant enhancement of antitumor efficacy of the therapy, accompanied by low toxicity, in various tumor models such as renal cell carcinoma [4] and advanced human ovarian cancer [5, 6] and pediatric cancers [7]. Topotecan, a topoisomerase I inhibitor, appears to be highly efficacious in animal models and more tolerable in human patients when administered for prolonged periods using metronomic dosing, without causing significant pharmacokinetic drug interactions. Regarding the efficacy of the combined treatment, our group recently published promising data in a human TNBC metastatic model [2]. Indeed, the potential therapeutic impact and the molecular mechanisms of topotecan administered in a continuous low-dose metronomic manner, either alone or in concurrent combination with pazopanib in both triple-negative primary and metastatic breast cancer models was evaluated. Our study showed the remarkable efficacy of the metronomic topotecan and pazopanib treatment combination in the metastatic triple negative breast cancer model causing significantly prolonged survival times of mice, associated with a significant decrease in tumor vascularity, proliferative index, and induction of apoptosis [2]. Moreover, metronomic topotecan in vitro caused a significant antiproliferative and proapoptotic activities on both endothelial and TNBC cells (231LM-2/4 – highly metastatic variant subline derived from the human TNBC cell line MDA-MB-231), which were enhanced by pazopanib treatment [2]. Metronomic topotecan and pazopanib therapy strongly inhibited the expression of hypoxia-inducible factor 1-alpha (HIF1α) and its target gene the ATP-binding cassette sub-family G member 2 (ABCG2) in hypoxic TNBC cells, increasing the intracellular concentration of the active form of topotecan [2]. These findings suggest the possibility of future clinical evaluation of oral low dose metronomic topotecan in combination with pazopanib or other antiangiogenic drugs as a novel and potential therapeutic option for the treatment of elderly triple-negative breast cancer patients with advanced metastatic disease.
